# Supplementary material for: Estimating Mean Viral Load Trajectory From Intermittent Longitudinal Data and Unknown Time Origins
Source: Stat Med. 2025 Feb 25;44(5):e70033. doi: 10.1002/sim.70033 (PMC11851093; doi:10.1002/sim.70033)
Supplement: Supplementary file 1 — Matlab code, Supporting Information [file SIM-44-0-s001.zip › appendices.pdf]

# Estimating Mean Viral Load Trajectory from Intermittent Longitudinal Data and Unknown Time Origins: Appendices

## Appendix I: The E-step

We need to calculate terms such as

$$\mathbb{E}_{\Theta^{(t)}}\Big((\boldsymbol{y}_i-\boldsymbol{\theta})^T\boldsymbol{\Sigma}^{-1}(\boldsymbol{y}_i-\boldsymbol{\theta}) \mid x_{i,1}=j, D_i\Big). \tag{1}$$

Using $(\boldsymbol{y}_i-\boldsymbol{\theta})^T\boldsymbol{\Sigma}^{-1}(\boldsymbol{y}_i-\boldsymbol{\theta}) = \text{trace}(\boldsymbol{\Sigma}^{-1}(\boldsymbol{y}_i-\boldsymbol{\theta})(\boldsymbol{y}_i-\boldsymbol{\theta})^T)$, and the additive property of the trace and expectation functionals, (1) can be simplified to

$$\text{trace}\{\boldsymbol{\Sigma}^{-1}\mathbb{E}_{\Theta^{(t)}}(\boldsymbol{y}_i\boldsymbol{y}_i^T \mid x_{i,1}=j, D_i)\} - 2\text{trace}\{\boldsymbol{\Sigma}^{-1}\boldsymbol{\theta}\mathbb{E}_{\Theta^{(t)}}(\boldsymbol{y}_i^T \mid x_{i,1}=j, D_i)\} + \boldsymbol{\theta}^T\boldsymbol{\Sigma}^{-1}\boldsymbol{\theta}. \tag{2}$$

Let $\boldsymbol{y}_{i|j}^T = [(\boldsymbol{y}_{i|j}^{\text{obs}})^T, (\boldsymbol{y}_{i|j}^{\text{unobs}})^T]$ be partitioned into its observed and unobserved parts for the case $x_{i,1}=j$, that is, $\boldsymbol{y}_{i|j}^{\text{obs}} = (y_{i,j}, y_{i,j+\Delta_{i1}}, \ldots, y_{i,j+\Delta_{im_i-1}})^T$ and $\boldsymbol{y}_{i|j}^{\text{unobs}}$ is the rest of the vector. Similarly, let $(\boldsymbol{\theta}^{(t)})^T = [(\boldsymbol{\theta}_{i|j}^{(t)\text{obs}})^T, (\boldsymbol{\theta}_{i|j}^{(t)\text{unobs}})^T]$ and

$$\boldsymbol{\Sigma}^{(t)} = \begin{pmatrix} \boldsymbol{\Sigma}_{i|j}^{(t)\text{obs}} & \boldsymbol{\Sigma}_{i|j}^{(t)\text{obs,unobs}} \\ (\boldsymbol{\Sigma}_{i|j}^{(t)\text{obs,unobs}})^T & \boldsymbol{\Sigma}_{i|j}^{(t)\text{unobs}} \end{pmatrix}$$

be the corresponding partition of the current (after iteration $t$) estimates of the mean and variance functions of $\boldsymbol{y}_i$. The expectations in (2) should be calculated as the expectation of $\boldsymbol{y}_i$ conditional on $\boldsymbol{y}_{i|j}^{\text{unobs}}$ using the probability law:

$$\boldsymbol{y}_{i|j} \equiv \begin{pmatrix} \boldsymbol{y}_{i|j}^{\text{obs}} \\ \boldsymbol{y}_{i|j}^{\text{unobs}} \end{pmatrix} \sim N\left(\begin{pmatrix} \boldsymbol{\theta}_{i|j}^{(t)\text{obs}} \\ \boldsymbol{\theta}_{i|j}^{(t)\text{unobs}} \end{pmatrix}, \begin{pmatrix} \boldsymbol{\Sigma}_{i|j}^{(t)\text{obs}} & \boldsymbol{\Sigma}_{i|j}^{(t)\text{obs,unobs}} \\ (\boldsymbol{\Sigma}_{i|j}^{(t)\text{obs,unobs}})^T & \boldsymbol{\Sigma}_{i|j}^{(t)\text{unobs}} \end{pmatrix}\right). \tag{3}$$

Using properties of the multivariate normal distribution:

$$\begin{aligned} \boldsymbol{e}_{i|j}^{(t)} &\equiv \mathbb{E}_{\Theta^{(t)}}(\boldsymbol{y}_{i|j}^{\text{unobs}} \mid x_{i,1}=j, \boldsymbol{y}_{i|j}^{\text{obs}}) = \boldsymbol{\theta}_{i|j}^{(t)\text{unobs}} + \big(\boldsymbol{\Sigma}_{i|j}^{(t)\text{obs,unobs}}\big)^T(\boldsymbol{\Sigma}_{i|j}^{(t)\text{obs}})^{-1}(\boldsymbol{y}_{i|j}^{\text{obs}} - \theta_{i|j}^{(t)\text{obs}}) \\ V_{i|j}^{(t)} &\equiv \text{Var}_{\Theta^{(t)}}(\boldsymbol{y}_{i|j}^{\text{unobs}} \mid x_{i,1}=j, \boldsymbol{y}_{i|j}^{\text{obs}}) = \boldsymbol{\Sigma}_{i|j}^{(t)\text{unobs}} - \big(\boldsymbol{\Sigma}_{i|j}^{(t)\text{obs,unobs}}\big)^T(\boldsymbol{\Sigma}_{i|j}^{(t)\text{obs}})^{-1}\big(\boldsymbol{\Sigma}_{i|j}^{(t)\text{obs,unobs}}\big)^T \end{aligned} \tag{4}$$

From (4) we obtain the terms for (2):

$$\boldsymbol{y}_{i|j}^{(t)} = \pi_{i|j} \circ \mathbb{E}_{\Theta^{(t)}}(\boldsymbol{y}_{i|j} \mid x_{i,1}=j, D_i) = \pi_{i|j} \circ [(\boldsymbol{y}_{i|j}^{\text{obs}})^T, (\boldsymbol{e}_{i|j}^{(t)})^T]^T, \tag{5}$$

and

$$\mathbf{C}_{i|j}^{(t)} = \pi_{i|j} \circ \mathbb{E}_{\Theta^{(t)}}(\boldsymbol{y}_{i|j}\boldsymbol{y}_{i|j}^T \mid x_{i,1}=j, D_i) = \pi_{i|j} \circ \begin{pmatrix} \boldsymbol{y}_{i|j}^{\text{obs}}(\boldsymbol{y}_{i|j}^{\text{obs}})^T & \boldsymbol{y}_{i|j}^{\text{obs}}(\boldsymbol{e}_{i|j}^{(t)})^T \\ \boldsymbol{e}_{i|j}^{(t)}(\boldsymbol{y}_{i|j}^{\text{obs}})^T & V_{i|j}^{(t)} + \boldsymbol{e}_{i|j}^{(t)}(\boldsymbol{e}_{i|j}^{(t)})^T \end{pmatrix}, \tag{6}$$

where $\pi_{i|j}$ rearrange the components to the original indexing.

## Appendix II: Linear covariance implementation

Consider a covariance matrix with a linear structure $\mathbf{\Sigma}(\boldsymbol{\beta}) = \sum_{j=1}^{J} \beta_j B_j$. That is, $\mathbf{\Sigma}(\boldsymbol{\beta})$ is a linear combination of $J$ known symmetric matrices $B_j$ ($j = 1, \dots, J$). Recall that at the $t+1$-th iteration, after computing $\boldsymbol{\theta}^{(t+1)}$, we have $\boldsymbol{Y}^{(t+1)} = \frac{1}{n} \sum_{i=1}^{n} \sum_{j=1}^{d-1} E_{ij}^{(t)} \boldsymbol{Y}^{(t)}_{\boldsymbol{\theta}^{(t+1)},i|j}$; see (**??**)-(**??**)). Given the above linear structure, our goal is to solve:

$$\min_{\boldsymbol{\beta}} \log|\mathbf{\Sigma}| + \mathrm{Tr}\left(\mathbf{\Sigma}^{-1}\boldsymbol{Y}^{(t+1)}\right) \quad s.t. \quad \mathbf{\Sigma} = \sum_{j=1}^{p} \beta_j \boldsymbol{B}_j.$$

Note that we isolated all terms which depend on $\mathbf{\Sigma}$ in (**??**), and divided by $\sum_{i=1}^{n} \sum_{j=1}^{d-1} E_{ij}^{(t)} = n$. Following [**?** ], the minimum point is found by repeatedly solving the linear equation $\boldsymbol{G}^{(\ell)}\boldsymbol{\beta}^{(\ell+1)} = \boldsymbol{g}^{(\ell)}$, where:

$$\boldsymbol{G}^{(\ell)} \in \mathbb{R}^{J\times J}: \quad [\boldsymbol{G}^{(\ell)}]_{k_1 k_2} = \mathrm{Tr}\left[\left(\sum_{j=1}^{J} \beta_j^{(\ell)} \boldsymbol{B}_j\right)\boldsymbol{B}_{k_1}\left(\sum_{j=1}^{J} \beta_j^{(\ell)} \boldsymbol{B}_j\right)\boldsymbol{B}_{k_2}\right]$$

$$\boldsymbol{g}^{(\ell)} \in \mathbb{R}^{J}: \quad [\boldsymbol{g}^{(\ell)}]_{k_1} = \mathrm{Tr}\left[\left(\sum_{j=1}^{J} \beta_j^{(\ell)} \boldsymbol{B}_j\right)\boldsymbol{B}_{k_1}\left(\sum_{j=1}^{J} \beta_j^{(\ell)} \boldsymbol{B}_j\right)\boldsymbol{Y}^{(t+1)}\right],$$

initialized by $\boldsymbol{\beta}^{(0)}$, until convergence. The final estimate $\hat{\boldsymbol{\beta}}$ forms the covariance $\hat{\mathbf{\Sigma}} = \sum_{j=1}^{J} \hat{\beta}_j \boldsymbol{B}_j$.
